# UPLC-QE-Orbitrap-Based Cell Metabolomics and Network Pharmacology to Reveal the Mechanism of N-Benzylhexadecanamide Isolated from Maca (*Lepidium meyenii* Walp.) against Testicular Dysfunction

**DOI:** 10.3390/molecules28104064

**Published:** 2023-05-12

**Authors:** Kai-Yue Zhang, Chun-Nan Li, Nan-Xi Zhang, Xiao-Chen Gao, Jia-Ming Shen, Duan-Duan Cheng, Yue-Long Wang, Hui Zhang, Jing-Wei Lv, Jia-Ming Sun

**Affiliations:** Jilin Ginseng Academy, Changchun University of Chinese Medicine, Changchun 130117, China; zky960523@163.com (K.-Y.Z.); lcn1013@hotmail.com (C.-N.L.); nancy_8080@outlook.com (N.-X.Z.); gao_xiaochen@hotmail.com (X.-C.G.); sjm1836778759@163.com (J.-M.S.); chengduanduan123@163.com (D.-D.C.); wyuelong1994@foxmail.com (Y.-L.W.); zhanghui-8080@163.com (H.Z.)

**Keywords:** testicular dysfunction, N-benzylhexadecanamide, metabolomics, network pharmacology, mechanisms

## Abstract

Testicular dysfunction (TDF) is characterized by testosterone deficiency and is caused by oxidative stress injury in Leydig cells. A natural fatty amide named N-benzylhexadecanamide (NBH), derived from cruciferous maca, has been shown to promote testosterone production. Our study aims to reveal the anti-TDF effect of NBH and explore its potential mechanism in vitro. This study examined the effects of H_2_O_2_ on cell viability and testosterone levels in mouse Leydig cells (TM3) under oxidative stress. In addition, cell metabolomics analysis based on UPLC-Q-Exactive-MS/MS showed that NBH was mainly involved in arginine biosynthesis, aminoacyl-tRNA biosynthesis, phenylalanine, tyrosine and tryptophan biosynthesis, the TCA cycle and other metabolic pathways by affecting 23 differential metabolites, including arginine and phenylalanine. Furthermore, we also performed network pharmacological analysis to observe the key protein targets in NBH treatment. The results showed that its role was to up-regulate ALOX5, down-regulate CYP1A2, and play a role in promoting testicular activity by participating in the steroid hormone biosynthesis pathway. In summary, our study not only provides new insights into the biochemical mechanisms of natural compounds in the treatment of TDF, but also provides a research strategy that integrates cell metabolomics and network pharmacology in order to promote the screening of new drugs for the treatment of TDF.

## 1. Introduction

The establishment and maintenance of the male testicular microenvironment both depend on Leydig cells, the principal sites of androgen synthesis and secretion [1]. Thus, Leydig cell damage is associated with decreased fertility and erectile function, and is a significant contributing factor to testicular dysfunction (TDF) occurrence. Oxidative stress, a potential cause of Leydig cell damage, can induce interstitial cell damage via apoptosis, resulting in reduced testosterone levels that compromise fertility [2]. Therefore, the inhibition of cell apoptosis may prevent interstitial cell damage to thereby avert the development and progression of H_2_O_2_-induced testicular failure [3].

So far, natural bioactive compounds have been found to be the most promising anti-TDF drug candidates [4,5]. Notably, N-benzylhexadecanamide (NBH, C_23_H_39_NO, 345.5712 g/mol), the main active component of maca, possesses anti-inflammatory, antioxidant, neuroprotective and yang-strengthening activities [6]. Importantly, the results of our previous analysis demonstrated that NBH might enhance sexual function, since this compound was found to prevent oxidative stress-induced TDF by increasing androgenic hormone activity [7]. These results notwithstanding, the mechanisms and targets underlying the NBH alleviation of TDF remain unclear.

**Figure 1:**
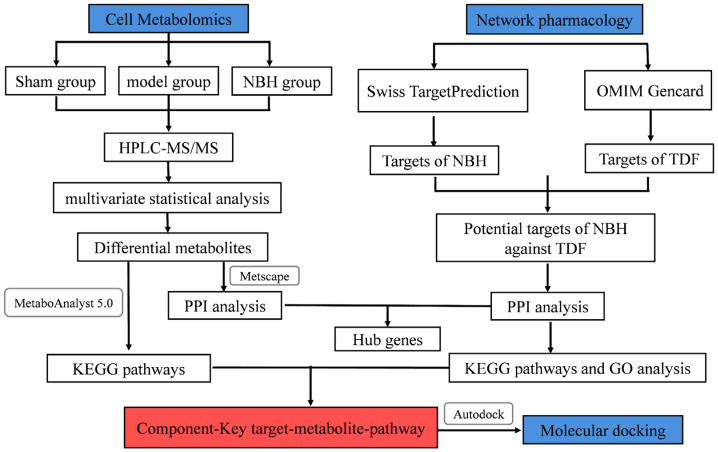
Integrated strategy flowchart.

Here, we conducted comprehensive metabolomics and network pharmacological studies to clarify the mechanism of action associated with the NBH alleviation of TDF. First, UPLC-QE-Orbitrap-MS non-target metabolomics was used to predict biomarkers and the related metabolic pathways associated with the NBH amelioration of oxidative stress effects in TM3 cells. Thereafter, the mechanism by which NBH alleviated TDF was identified using integrated network pharmacological methods. These results should provide reference data to facilitate future investigations of the origins of TDF, while also extending the scope of NBH clinical applications. The flowchart shown in Figure 1 outlines the research process used in this work.

## 2. Results

### 2.1. Different Maca Polar Extracts Affect TM3 Cell Activity

**Figure 2:**
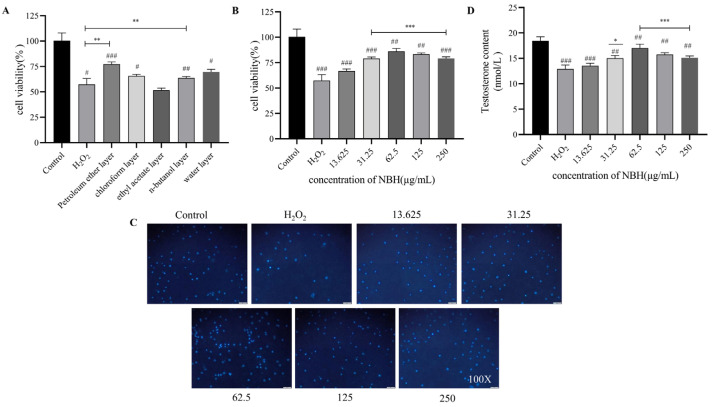
Effects of different polar extracts of maca on TM3 cell activity. (**A**) TM3 cell proliferation was measured using MTT in the presence of different concentrations of NBH. (**B**) A fluorescent microscopic image of DAPI-stained TM3 cells after NBH treatment (×100). (**C**) Testosterone was detected in TM3 cells via immunofluorescence assays. (**D**) ^#^ *p* < 0.05, ^##^ *p* < 0.01 and ^###^ *p* < 0.001 compared to the Sham group; * *p* < 0.05, ** *p* < 0.01 and *** *p* < 0.001 compared to the Model (H_2_O_2_) group.

The results showed that 93.15 g of petroleum ether layer, 92.97 g of chloroform layer, 50.66 g pf ethyl acetate layer, 343.31 g of n-butanol layer and 2452.91 g of water layer were obtained. MTT assays were conducted to determine the cellular-level activities of different polar extracts of maca. The results revealed that all maca extracts, except for the extract obtained using ethyl acetate, promoted the proliferation of Leydig cells, of which the petroleum ether extract possessed the strongest activity (as shown in Figure 2A below).

### 2.2. Identification of NBH

The petroleum ether extract was purified to obtain 3.16 mg of a white powder that was soluble in methanol. Testing of the powder via a modified bismuth potassium iodide test yielded a positive result that indicated that the compound contained nitrogen.

The results of ^1^H-NMR spectroscopy were as follows: (CDCl_3_, 500 MHz) δ (ppm); a group of benzene signals was detected and assigned to δ7.30 (1H, m, H-3′), δ7.35 (1H, m, H-4′), δ7.29 (1H, m, H-5′), δ7.35 (1H, m, H-6′), δ7.29 (1H, m, H-7′); δ4.47 was the benzylic position proton signal (2H, d, *J* = 5.6 Hz) and δ5.78 (1H, brs) was the nitrogen-connected proton signal. The long-chain hydrocarbon proton signals showed δ2.24 (2H, t, *J* = 7.6 Hz, H-2) δ1.68 (2H, m, H-3), δ1.31 (24H, m, H-4-H-15) and δ0.9 (3H, t, *J* = 7.6 Hz, H-16). According to the abovementioned data, the compound with the *m*/*z* value of 346.2788 was NBH and its spectrum is shown in Figure S1.

### 2.3. Effects of NBH on TM3 Cell Viability and Testosterone Content

Oxidative stress-induced testicular cell apoptosis and fibrosis are considered major causes of TDF. Notably, the MTT assay results showed a strong NBH treatment-induced reversal of H_2_O_2_-induced TM3 cell decline (Figure 2B), while the DAPI fluorescence results revealed that NBH increased the H_2_O_2_-induced fluorescence intensity. (Figure 2C). In addition, the H_2_O_2_-induced testosterone content was largely reversed by NBH treatment (Figure 2D). In the above-mentioned results, NBH has the strongest effects when the concentration is 62.5 μg/mL.

### 2.4. Metabolomics Analysis

#### 2.4.1. Analyzing System Stability and QC Sample Detection

**Figure 3:**
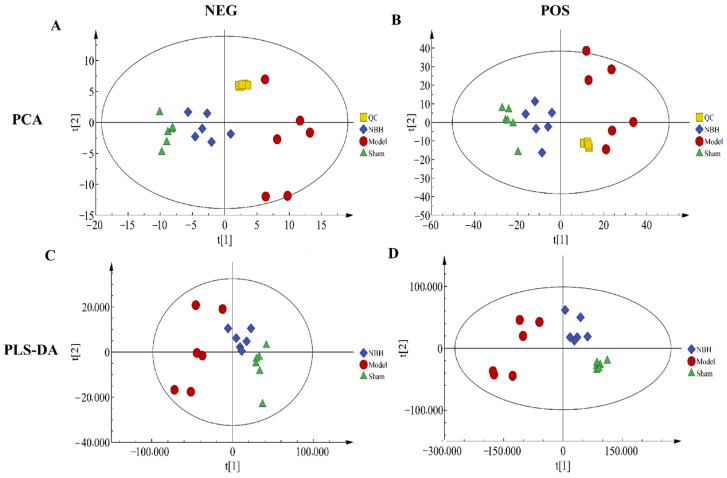
Score plots of QC, Sham, Model and NBH groups in NEG (**A**) and POS mode (**B**) (*n* = 6); PLS-DA scores plot for Sham, Model and NBH groups in NEG (**C**) and POS mode (**D**) (*n* = 6).

In Figure S2, representative base peak chromatograms (BPC) obtained in the positive (POS) and negative (NEG) ion modes for the three groups are shown. The results revealed the detection of 14,023 peaks in the POS ion mode and 12,631 peaks in the NEG ion mode. To remove noise, the relative standard deviation (RSD, CV) was applied to each single peak. Data standardization was achieved using internal standards (IS). After the NBH pretreatment of cells, the POS retained 8692 peaks and the NEG retained 6410 peaks. In order to ensure the stability and reliability of the system and the data, quality control (QC) samples were tested during the analysis process. Then, the PCA model was run on all samples. According to Figure 3, the QC samples showed good aggregation in the PCA score plot, thus suggesting stable experimental conditions from the first to the last sampling.

#### 2.4.2. Statistical Analysis of Multivariate Data

**Figure 4:**
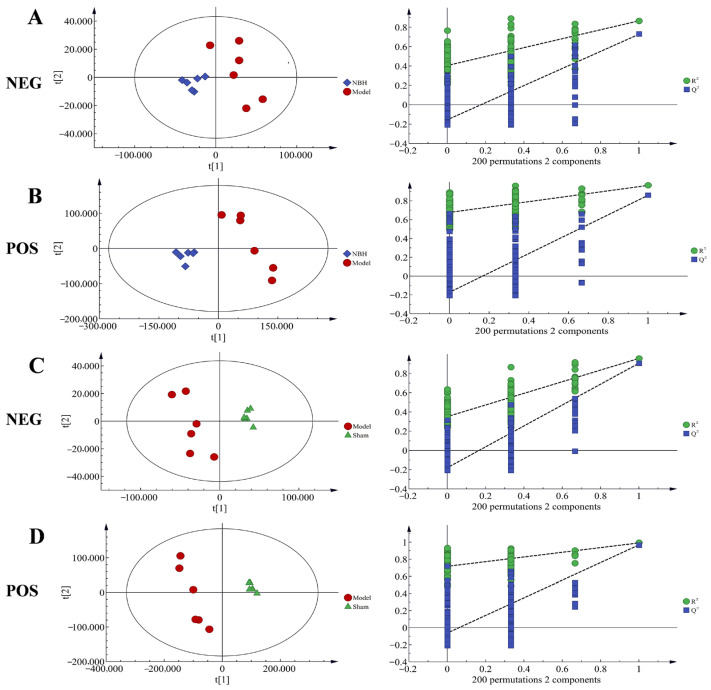
PLS-DA score plots of the Sham group, model group, and NBH-treated group based on negative ion and positive ion data. NBH-treated group vs model: negative ion (**A**) and positive ion (**B**); Model group vs. Sham group: negative ion (**C**) and positive ion (**D**).

PCA was used to analyze the metabolic fingerprint changes in the TM3 cells treated with NBH. According to the PCA score plot, the metabolic fingerprints changed over time. Using the PLS-DA model, we were able to differentiate between the Sham group and Model group results, as well as between the Model group and NBH group results. Based on the parameters of the model (Table 1), the PLS-DA was suitable for experiments. Using the PLS-DA model, the results for the NBH group were found to be significantly distinct from the Model group results in the two scanning models; meanwhile, the NBH group results were closer to the Sham group results than to the Model group results, indicating that NBH could restore TM3 cell homeostasis after oxidative damage. Across all PCA and OPLS-DA scores, a consistent trend of clear separation between the two comparison groups indicated that the NBH treatment altered TM3 cell metabolism (Figure 4).

#### 2.4.3. Biomarker Identification

**Figure 5:**
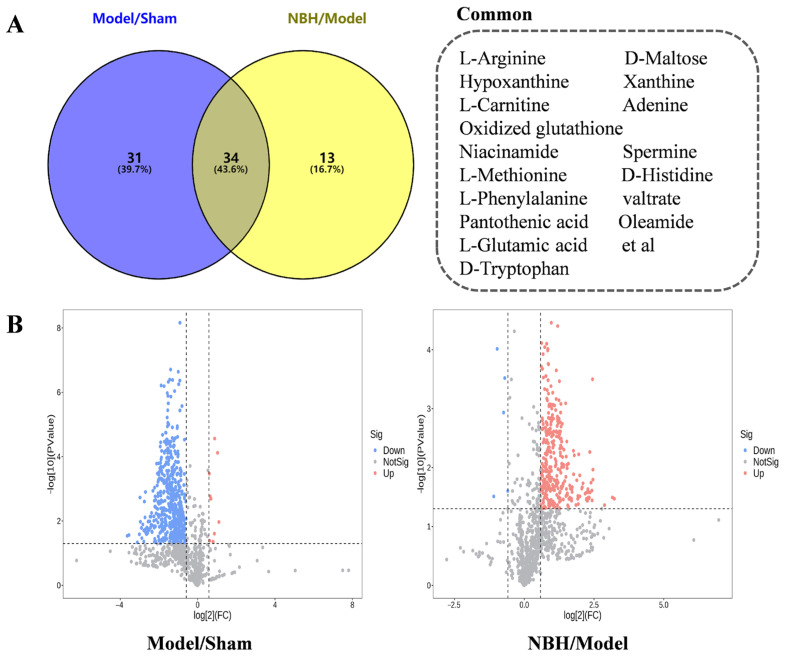
Model/Sham/Model Venn diagram (**A**); Volcano plot comparing NBH/Model and Model/Sham results (**B**). X and Y axes represent log_2_ fold change and −log_10_ *p*-value, respectively.

**Figure 6:**
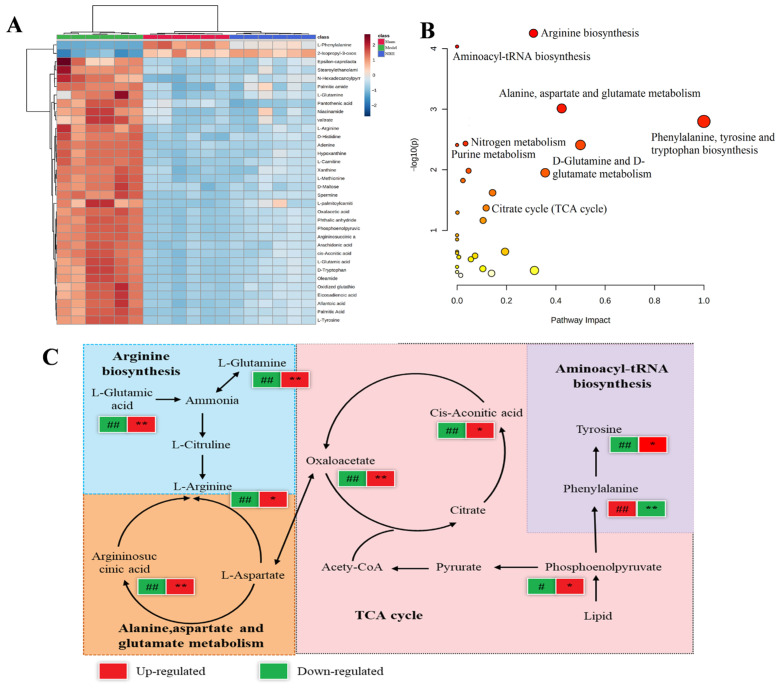
Heat map (**A**) showing changes in intensities of potential biomarkers; bubble plot (**B**) showing the response of the main NBH-perturbed TDF-related pathway; metabolic pathways of TDF in NBH-treated TM3 cells (**C**), ^#^ *p* < 0.05. ^##^ *p* < 0.01, the Model group versus the Sham group. * *p* < 0.05. ** *p* < 0.01, the NBH group versus the Model group.

The orthogonal projections to latent structures discriminant analysis (OPLS-DA) and *t*-test (*p* < 0.05) results led to the identification of metabolites with VIP > 1 that were subsequently selected as potential biomarkers. Analysis of the Model/Sham and NBH/Model comparison results led to the identification of 65 and 47 potential biomarkers, respectively, with 34 potential biomarkers shared between them (Figure 5A,B, Table 2). Taken together, these results indicated that NBH-induced TDF may be diagnosed using these 34 biomarkers, of which the expression levels of two of them were significantly down-regulated by the NBH treatment: 2-isopropyl-3-oxosuccinate and L-phenylalanine (Figure 6A).

#### 2.4.4. Metabolic Pathway Analysis

MetaboAnalyst was used to analyze the pathway enrichment based on the KEGG identifiers of the 34 potential biomarkers. The results revealed that the NBH treatment significantly altered the relevant metabolic pathways, including arginine biosynthesis, aminoacyl-tRNA biosynthesis, phenylalanine, tyrosine and tryptophan biosynthesis, the citrate cycle and others, as shown in the bubble plot (Figure 6B). Using a metabolomic approach, we identified and constructed the relevant metabolic pathways involved in the NBH alleviation of TDF (Figure 6C).

### 2.5. Network Pharmacology Analysis

#### 2.5.1. Analysis of Metabolic Targets Related to Potential Biomarkers

**Figure 7:**
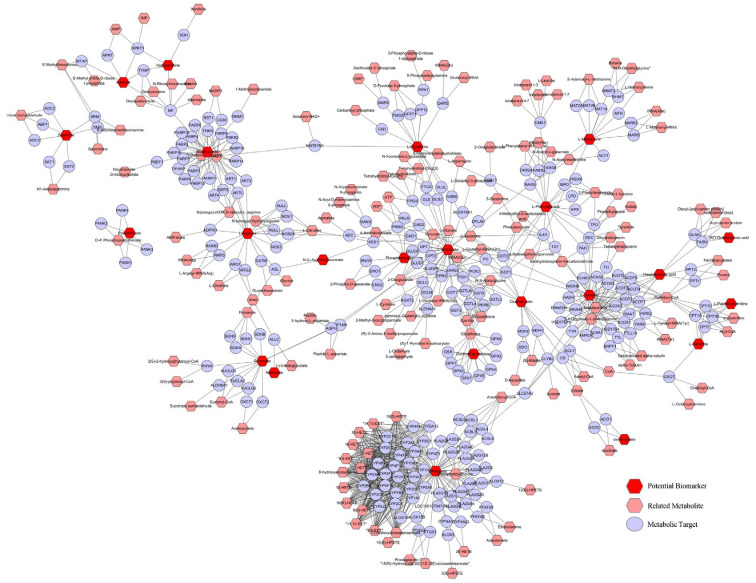
Metabolic targets are based on potential biomarkers.

Using Cytoscape, 133 metabolic targets were identified based on putative biomarkers (Figure 7).

#### 2.5.2. Targets Predicted by the Network That Are Related to the NBH Alleviation of TDF

**Figure 8:**
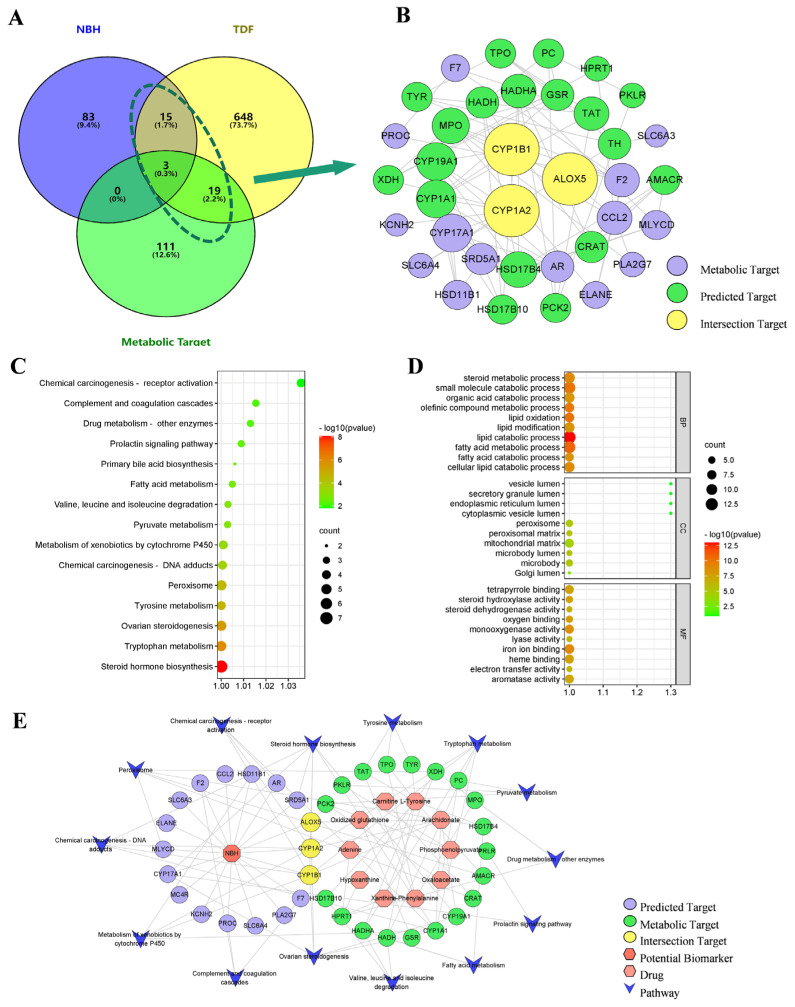
Predicted NBH targets and metabolic targets related to the alleviation of TDF are shown in the Venn diagram below (**A**). Construction of potential PPI network related to the NBH alleviation of TDF (**B**). Results of KEGG (**C**) and GO (**D**) pathway enrichment analyses for each cluster. The final integrated “Potential Biomarkers–Targets-Pathways” network (**E**).

Using SwissTargetPrediction, 101 predicted NBH targets were identified, as well as 685 TDF targets that were retrieved from GeneCards and OMIM databases. In addition to metabolic targets, 37 targets based on the NBH alleviation of TDF were selected from the network crossover points (Figure 8A).

#### 2.5.3. PPI Network Analysis

Based on the highest confidence level (0.9) results defined for the race, 38 predicted targets were identified that were subsequently analyzed using the String database. The results of this analysis were then imported into Cytoscape in order to build the PPI network (Figure 8B). Three key nodes, including CYP1A2, CYP1B1 and ALOX5, that were associated with the NBH alleviation of TDF were identified.

#### 2.5.4. Analysis of the ‘Potential Biomarker-Target-Pathway’ Network

KEGG pathway and GO enrichment analyses were performed on 38 central genes identified using the MetScape database in order to elucidate the biological processes associated with key targets (Figure 8C,D). The main pathways that were identified included steroid hormone biosynthesis, ovarian steroid synthesis, pyruvate metabolism and the prolactin signaling pathways. Additionally, the identified GO terms that were enriched in the relevant groups included cellular lipid catabolism, steroid metabolism and galactosyltransferase activity. Thereafter, 10 potential biomarkers, 22 metabolic targets, 18 predicted targets and 14 pathways were combined to develop a potential biomarker–target pathway network (Figure 8E). Within this network, CYP1A2, CYP1B1 and ALOX5 targets were detected at intersections between the metabolic targets and predicted targets, thus suggesting that these targets play key roles in the effect of NBH against testosterone deficiency.

#### 2.5.5. A Molecular Docking Results

**Figure 9:**
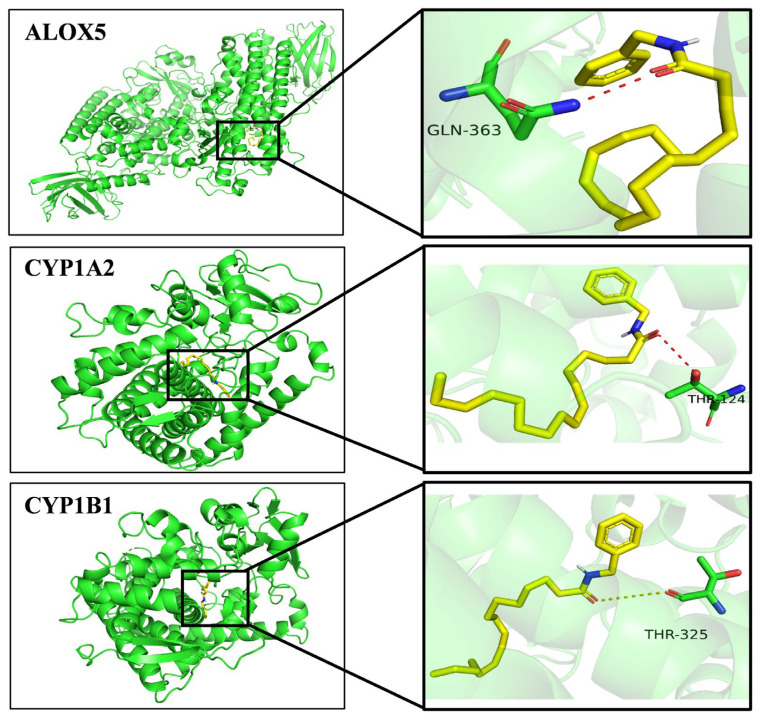
Docking diagram between NBH and hub genes (G).

As a result of network and metabolomics analyses, CYP1A2, CYP1B1 and ALOX5 were identified as the most relevant proteins involved in the NBH alleviation of TDF. Based on these results, autodock was used to dock the 3D structures of these proteins to the NBH active compound. In Figure 9, the binding activity of NBH to CYP1A2 was −8.6 kcal/mol, to CYP1B1 it was was −8.2 kcal/mol, and to ALOX5 it was −5.8 kcal/mol. The results showed that the predicted target exhibited a stable structure similar to that of the metabolic target, thus indicating that the prediction results were highly accurate.

**Figure 10:**
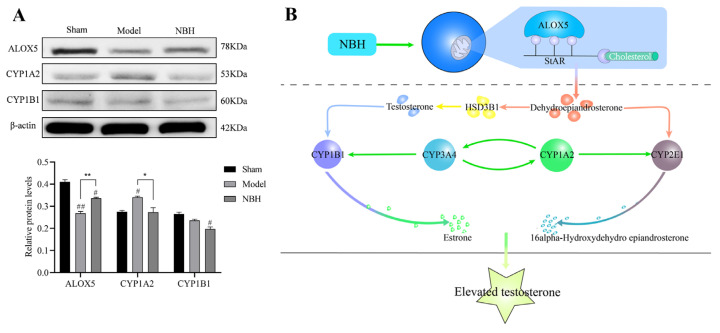
The expression of ALOX5, CYP1A2 and CYP1B1 was assessed by Western blot (**A**). Diagram of the mechanism of action of NBH on hug genes (**B**). ^#^ *p* < 0.05 and ^##^ *p* < 0.01 compared to the Sham group; * *p* < 0.05 and ** *p* < 0.01 compared to the Model group.

To confirm the three key metabolic enzymes predicted above, we performed Western blotting analysis. After 62.5 g/mL of NBH treatment, the CYP1A2 levels in TM3 cells were significantly decreased (*p* < 0.05) and the ALOX5 levels were significantly increased (*p* < 0.01). The results demonstrated that NBH could control the expression of CYP1A2 and ALOX5, which might have a major impact on the metabolism of TM3 cells (Figure 10A).

## 3. Discussion

Testosterone is known to exert various positive effects on human health [8,9]. However, increased age and social stress can trigger the development of testosterone deficiency that can lead to the loss of skeletal muscle, to decreased exercise capacity, and to increased depression and sperm inactivation. Testosterone is produced by Leydig cells that resemble TM3 cells isolated from mouse testes [10,11]. Using an in vitro TDF model based on H_2_O_2_-induced oxidative damage to TM3 cells, the results of this study showed that NBH enhanced TM3 cell viability under oxidative stress conditions and increased testosterone levels. Optimal effects were observed for a concentration of NBH of 62.5 μg/mL. Subsequently, the metabolomics approach of LC-MS was used to further investigate the potential mechanisms of NBH for the alleviation of TDF in TM3 cell models. Based on the changes of cell metabolites induced by H_2_O_2_, we compared the results obtained in the sham operation group, the model group and the NBH group to study the effect of NBH on the nodes related to TM3 proliferation and testosterone secretion. Notably, the cellular metabolic profile changed significantly after NBH treatment, as reflected by changes in the levels of 34 metabolites, including arachidonic acid, arginine and glutamate, among others. These results thus indicated that NBH treatment induced complex changes in metabolic processes, as reflected by changes in the expression of these potential biomarkers, which may be helpful for diagnosing TDF. Furthermore, the functions of these biomarkers were mainly related to arginine biosynthesis, aminoacyl-tRNA biosynthesis and the tricarboxylic acid (TCA) cycle, suggesting that multiple metabolic pathways may regulate NBH anti-TDF therapeutic effects. Therefore, based on the results of the network pharmacological analysis, potential therapeutic targets related to the NBH alleviation of TDF were identified.

Several studies have linked amino acid metabolism to testosterone deficiency [12,13], with amino acids potentially playing a dual etiological role in the development of testosterone deficiency and related disorders [14]. On the one hand, the results of this study revealed increased levels of amino acids, such as arginine, tryptophan, glutamic acid, pantothenic acid and others, in the NBH group, which is consistent with our NMR metabolomics findings [15]. Arginine biosynthesis, which is necessary for sperm maturation within the epididymis, is promoted by NBH treatment, which supports sperm production for improved male fertility. By contrast, tryptophan acts as an antioxidant in the body, while also scavenging reactive oxygen species and reactive nitrogen species, and enhancing the body’s antioxidant capacity. Meanwhile, the synthesis and secretion of testosterone are regulated by glutamate, whereby up-regulated glutamine and glutamate production supply is necessary for testosterone synthesis and secretion during testicular cell proliferation. Furthermore, the up-regulation of pantothenate promotes histone acetylation and the activation of cell growth-related genes by elevating acetyl-CoA via the pantothenate and CoA biosynthetic pathways [16,17]. When cells are under oxidative stress, the release of arachidonic acid from membrane phospholipids occurs, which allows it to combine with acetyl-CoA to generate acetyl-CoA ester, which is metabolized by the cyclooxygenase pathway [18,19].

Interestingly, in the NBH group, phosphoenolpyruvate and oxaloacetic acid production were significantly up-regulated and glycolysis was impeded compared to the corresponding Model group activity levels. Phosphoenolpyruvate is an intermediate product of glycolysis and gluconeogenesis. The up-regulation of phosphoenolpyruvate production indicates that the spontaneous glycolysis process provides energy for cell proliferation, which generates pyruvate that is transported to the inner mitochondrial membrane [20,21]. Thereafter, the production of acetyl-CoA is significantly up-regulated, which links the TCA cycle to a variety of other metabolic pathways. Importantly, the biomarkers and network pharmacology analysis results obtained here identified three closely related NBH targets, CYP1A2, CYP1B1 and ALOX5, of which CYP1A2 and CYP1B1 are members of the CYP450 family, which converts cholesterol to testosterone. When cholesterol is transferred to mitochondrial inner membranes, P450scc cleaves cholesterol side chains to release pregnenolone, a steroid that is converted into testosterone [22]. In addition, P450scc participates in arachidonic acid metabolism by converting hydroperoxide species into oxo-metabolites.

Arachidonic acid 5-lipoxygenase (ALOX5) is an important drug target within the arachidonic acid metabolic pathway [23,24]. Arachidonic acid can activate ALOX5 to generate 5 (S)-HPETE, while also affecting the expression of steroidogenic acute regulatory (StAR) protein, a key protein involved in testosterone synthesis [25]. Mechanistically, this effect depends on arachidonic acid metabolism, an important metabolic pathway that triggers the entry of cytoplasmic 5 (S)-HPETE into the nucleus. Once in the nucleus, HPETE regulates DNA transcription and boosts the expression of StAR, a rate-limiting protein within the steroid synthesis pathway. This event thereby prompts StAR to transport cholesterol from the mitochondrial outer membrane to inner mitochondrial membranes, thus demonstrating the important role of arachidonic acid metabolism in cholesterol metabolism. As a key enzyme involved in arachidonic acid metabolism, ALOX5 exhibits strong catalytic activity after the Ser663 residue within the enzyme is phosphorylated [26]. Notably, NBH acts directly on Ser663 of ALOX5 to regulate StAR activity and thereby increase the cholesterol transport from mitochondria.

In the steroid hormone biosynthesis signaling pathway, after cholesterol enters mitochondria, it undergoes a series of enzymatic reactions to generate the testosterone precursor dehydroepiandrosterone (DHEA) and estrone [27]. DHEA is metabolized by three enzymes, CYP1A2, CYP2E1 and CYP3A4, which synergistically act to produce 16α-hydroxydehydro epiandrosterone (Figure 10B). The molecular docking results showed that NBH directly binds to characteristic amino acid residues within the CYP1A2 active center to competitively inhibit the activity of this enzyme. In turn, CYP1A2 inhibition reduces the production of 16α-hydroxydehydro epiandrosterone to thereby avoid the excessive consumption of the testosterone precursor DHEA [28], thus making it available for use as a raw material for testosterone synthesis. At the same time, NBH may directly inhibit CYP1B1 activity by binding to key active center amino acid residues to reduce the conversion of testosterone to estrone, thereby increasing the level of testosterone in TM3 cells. To identify the above three key metabolic enzymes, we performed protein blot analysis. It was found that NBH significantly promoted the expression of ALOX5, and decreased the expression of CYP1A2, thereby increasing testosterone levels.

## 4. Materials and Methods

### 4.1. Reagents and Material

Whole maca plants (220119) were obtained from the Changchun University of Chinese Medicine Slicing Factory, Ltd. (Changchun, China). Leydig (TM3) murine cells were purchased from the Chinese Academy of Sciences (Beijing, China). Testosterone ELISA kits were purchased from Changchun Bestgene Biotechnology (Changchun, China). RPMI-1640 (31800022) and fetal bovine serum (12250) was obtained from Gibco (Grand Island, CA, USA). Methanol, acetonitrile, formic acid and H_2_O were all of high-performance liquid chromatography (HPLC) grade (Fairfield, OH, USA), while all the remaining reagents were of analytical grade.

### 4.2. Plant Names

*Lepidium peruvianum* Chacon, also called ‘*Lepidium meyenii* Walp.’ or ‘maca’, is a medicinal plant that is described online at http://www.theplantlist.org/ (accessed on 18 July 2022). This species, which belongs to the family Cruciferae, genus Lepidium, is widely distributed in high-altitude areas.

### 4.3. Preparation of Maca Extract

In order to prepare each extract, dried maca roots (7.5 kg) were ground into coarse powder (40 mesh). Next, that pow was extracted under reflux with 10 times of 70% ethanol for 1 h, the final extracts were combined, and the ethanol was recovered under reduced pressure to obtain a dry extract. A thick paste was next prepared by extracting the filtrate using petroleum ether and then chloroform, ethyl acetate or n-butanol to produce a paste dried under vacuum.

### 4.4. Isolation and Identification of NBH

After dissolving 50 g of petroleum ether dry extract in methanol, it was centrifuged at 12,000 rpm for 20 min. The supernatant was filtered through a 0.45 μm membrane. The filtrate was passed through Sephadex LH-20, repeatedly collected via elution with methanol, purified in a semi-preparative liquid phase, and the purified samples were subjected to comprehensive analysis using 6520 Accurate-Mass Q-TOF MS and a 600 MHz Avance III NMR Spectrometer to identify the chemical composition of the final extract. 

### 4.5. Cell Culture

TM3 cells were cultured in RPMI-1640 containing 10% FBS, 100 U/mL of penicillin and 100 μg/mL of streptomycin at 37 °C in a humidified environment with 5% CO_2_. All cells incubations were performed under these conditions unless otherwise specified.

### 4.6. Cell Viability Assay

TM3 cells (2 × 10^4^ cells/well) were cultured for 24 h in 96-well plates. Thereafter, the cells were pretreated with a series of NBH concentrations (13.625, 31.25, 62.5, 125 or 250 µg/mL) for 24 h, then were exposed to H_2_O_2_ for 4 h to establish oxidative damage conditions. Next, 10 μL of activated MTT was added to each well, followed by the incubation of plates for 4 h. Thereafter, the absorbance value at 490 nm was measured for each well.

### 4.7. Testosterone Levels Testing

TM3 cells were cultured in 6-well plates at 3 × 10^5^ cells/well for 24 h. Next, cells were treated with NBH at the abovementioned concentrations, and the testosterone contents of the culture supernatants in wells were detected using an ELISA kit.

### 4.8. Metabolomics Analysis

#### 4.8.1. Preparation of Cell Samples for Sham, Model and NBH Groups

TM3 cells were seeded into 100 mm diameter petri dishes at a density of 1 × 10^7^ cells per dish, then cultured for 24 h with RPMI-1640 (Sham group), H_2_O_2_ (Model group) or H_2_O_2_ + 62.5 g/mL NBH (NBH group). After culture, cells were washed twice with pre-cooled PBS followed by trypsin digestion. In order to obtain cell pellets, cells were suspended in PBS, transferred to 1.5 mL microcentrifuge tubes, then the tubes were centrifuged at 2500× *g* for 5 min at 4 °C and the supernatants were removed [29,30]. Prior to analysis, the cell pellets were stored at −80 °C.

#### 4.8.2. Metabolite Extraction

First, 1000 µL of solution containing 1 µg/mL (methanol: water = 4:1) was added to each cell pellet. Then, the samples were vortexed for 30 s, homogenized at 5000 rpm for 4 min and sonicated for 6 min in an ice bath. After homogenization and ultrasound treatment, the samples were incubated for 1 h at −20 °C and then centrifuged for 15 min at 4 °C at 13,000× g. For LC/MS analysis, the supernatants were transferred to new glass vials. Quality control (QC) samples were prepared by mixing equal volumes of sample supernatants.

#### 4.8.3. LC–MS/MS Analysis

Separation was carried out using an UltiMate 3000 HPLC system and an Accucore^TM^ C_18_ column (2.1 × 100 mm, 1.7 μm, Waters) maintained at 25 °C. The proportions of formic acid (A) and acetonitrile (B) averaged 0.1% (*v*/*v*) in the mobile phase. Based on the elution gradient, the analysis was conducted as follows: 0–4 min, 2–12% B; 4–10 min, 12–24% B; 10–20 min, 24–100% B; and 20–28 min, 100% B. The autosampler operated at 4 °C with an injection volume of 10 μL and a flow rate of 0.3 mL/min.

As an important feature of the UPLC-QE-Orbitrap-MS system, positive ion (ESI^+^) and negative ion (ESI^−^) electrospray ion sources were used within the scan range of 66.7 to 1000 *m*/*z*. The MS source parameters were set as follows: 35 irregular units of jacket gas flow, 15 irregular units of auxiliary gas flow and 1 irregular unit of purge gas flow. In the MS/MS mode, the impact energy was set to 10%, 20% and 40% of the normalized collision energy. Xcalibur software (version 2.2.42, Thermo Fisher Scientific, Waltham, MA, USA) was used to record and analyze data.

#### 4.8.4. Multivariate Statistical Analysis

##### Data Processing and Biomarker Selection

Compound Discoverer (CD version 3.0, Thermo Scientific) was used to obtain detailed information about each peak within the LC–MS raw data. The results were analyzed using SIMCA software (version 14.1, Umetrics, Méo, Sweden). To examine the cell metabolite differences among the control and experimental groups, a simplified discriminant analysis (PLS-DA) was conducted. Then, the quality of the PLS-DA model was evaluated based on R^2^Y and Q^2^ measurements. The use of VIP (VIP > 1) projection and value change (FC > 2) settings resulted in significantly improved results compared to the results obtained using other settings. GraphPad Prism 6.0 software (La Jolla, CA, USA) was used to determine the significance levels for the differences found between groups. Metabolites with differences that reached statistical significance (*p* < 0.05) were selected as biomarkers.

Cell metabolites were identified by matching the actual M/S data with the MS/MS data for endogenous metabolites obtained from The Human Metabolome Database (HMDB, https://www.hmdb.ca (accessed on 25 August 2022)) and Metlin (https://metlin.scripps.edu (accessed on 25 August 2022)), while MetaboAnalyst 5.0 (https://www.metaboanalyst.ca/MetaboAnalyst/ (accessed on 25 August 2022)) was used to identify the associated metabolic pathways.

##### Hierarchical Cluster Analysis and Volcano Plots

To better understand the overall biomarker profiles, volcano plots were created that listed the biomarkers and controls in ascending order by significance. Next, the Euclidean interval was calculated, and the values were ranked in order and used to spatially arrange large numbers of potential biomarkers within large aggregates, which were displayed as a heat map. Thereafter, Pearson coefficients were calculated to determine the correlation coefficients for each pair of variables. Then, the relative statistical significance values for the relative difference values obtained for the potential biomarkers between groups were presented as volcano plots.

##### Metabolic Pathway Analysis

All biomarker-related processes were comprehensively identified using the Kyoto Encyclopedia of Genes and Genomes (KEGG), a database combining genomic sequencing and other high-throughput data [31]. Thereafter, the identified processes were further analyzed using structural and topological analyses in order to identify the main molecular pathways associated with the NBH alleviation of TDF in TM3 cells. This method is intuitive and permits the effective visualization of the results.

### 4.9. Network Pharmacology Study

#### 4.9.1. Analysis of Metabolic Targets

Biomarker data were uploaded onto MetScape (Cytoscape software plug-in) in order to transform and create ‘potential biomarkers–target’ networks.

#### 4.9.2. NBH Network Prediction Target Selection

A practical strategy based on data linking and document extraction was used to predict NBH targets. The most likely biological target of NBH was predicted using SwissTargetPrediction (http://www.swisstargetprediction.ch/ (accessed on 19 January 2023)). Targets associated with TDF were found using the Online Mendelian Inheritance in Man (OMIM, https://www.omim.org/about (accessed on 22 January 2023)) and GeneCards (https://www.genecards.org/ (accessed on 22 January 2023)) databases. The common predicted targets shared by the NBH and TDF datasets were retained and other targets were removed using uniprot (https://www.uniprot.org/ (accessed on 25 January 2023)).

#### 4.9.3. Construction of a Network of Interactions between TDF-Related Proteins and NBH-Induced Proteins

Protein–protein interaction (PPI) analysis was conducted using the String database (https://string-db.org/, version 11.0 (accessed on 26 January 2023)). Hub gene data and analysis data were filtered to the same degree.

#### 4.9.4. GO Process and KEGG Pathway Enrichment Analysis

GO biological process analysis and KEGG pathway enrichment analysis are common methods for analyzing genes shared by different networks. Here, high-throughput bioinformatics-based functional annotation and the enrichment analysis of hub genes were conducted. Then, gene functions were identified based on total scores (*p* < 0.05). The results of the GO and KEGG analyses were then used to select and depict the NBH target-related metabolic pathways as target–pathway networks.

#### 4.9.5. Molecular Docking and Construction of a ‘Potential Biomarker–Target-Pathway’ Network

A comprehensive potential biomarker–target–pathway network was constructed by combining potential biomarker–target pairs, PPI results and target–pathway networks. This network enabled the further exploration of deeper links between the upstream and downstream networks. Ultimately, AutoDock Vina was used to systematically evaluate the drug functions based on the molecular recognition results. From the Research Collaboratory for Structural Bioinformatics (RCSB) Protein Data Bank (PDB) database, the crystal structures of the target proteins CYP1A2 (PDB ID: 2HI4), CYP1B1 (PDB ID: 6IP5) and ALOX5 (PDB ID: 6N2W) were accessed and downloaded. Active compounds (ligands) and target proteins (receptors) were processed to reduce energy, remove receptor water molecules, add polar hydrogen atoms, add charges and add magnetic fields prior to docking. Unless otherwise stated, all parameters were set to default values.

#### 4.9.6. Western Blot Assay

As described earlier, TM3 cells were cultured in 6-well plates. Cells were harvested after 24 h with ice-cold RIPA lysis buffer containing protease inhibitors and incubated at 4 °C for 60 min. After centrifugation at 13,532× *g* and 4 °C for 5 min, the cell debris was precipitated. A Bradford assay kit was used to quantify the protein concentration in the supernatant. Overall, 20 μg of protein was loaded onto polyacrylamide gels for sodium dodecyl sulfate electrophoresis (SDS-PAGE) in each well and mixed with 5 g of SDS loading buffer. Separated proteins were transferred onto a PVDF membrane (Millipore, IPVH00010, Billerica, MA, USA) and analyzed using different primary antibodies (anti-CYP1A2, anti-CYP1B1, anti-ALOX5 and anti-β-actin). In accordance with the manufacturer’s instructions, the Odyssey Infrared Imaging System (LI-COR Biosciences, Nebraska, NE, USA) was used to visualize the target proteins. During the experiments, the target proteins were expressed in triplicate, independently, and the expression was normalized to that of β-actin.

### 4.10. Statistical Analysis

At least three independent experiments were conducted. The results are expressed as the mean ± SD. Microsoft Excel and GraphPad Prism 8.0.2 were used for data collection and analysis. One-way analysis of variance (ANOVA) was performed to assess the significance of the NBH dose-dependent treatment effects.

## 5. Conclusions

In this work, the protective effect of NBH against TDF was systematically examined using LC–MS-based metabolomic and network pharmacology analyses. Ultimately, the metabolomic analysis identified 34 significantly altered biomarkers in NBH-treated versus untreated oxidatively stressed TM3 cells that were related to arginine biosynthesis, aminoacyl-tRNA biosynthesis, etc., while three core intersection targets, CYP1A2, CYP1B1 and ALOX5, were identified using network pharmacology. Next, the binding interactions between the three core intersection target proteins and active NBH-derived compounds were visualized at the molecular level using molecular docking analysis. To identify the above three key metabolic enzymes, we performed protein blot analysis. It was found that NBH significantly promoted the expression of ALOX5, and decreased the expression of CYP1A2, thereby increasing testosterone levels. These results should enhance our understanding of the regulatory mechanism underlying the NBH alleviation of TDF to facilitate the further development of NBH and its related compounds for potential use as supplements for alleviating TDF.

## Figures and Tables

**Table 1:** Analyses of different models with their parameters.

Model	Mode	Type	A	N	R^2^X	R^2^Y	Q^2^	Title
Model 1	Neg	PCA	4	24	0.775	-	-	Total
Model 2	Pos	PCA	4	24	0.696	-	-	Total
Model 3	Neg	PLS-DA	3	18	0.888	0.676	0.301	Total
Model 4	Pos	PLS-DA	3	18	0.536	0.903	0.315	Total
Model 5	Neg	PLS-DA	2	12	0.86	0.866	0.727	NBH/Model
Model 6	Pos	PLS-DA	2	12	0.5	0.964	0.858	NBH/Model
Model 7	Neg	PLS-DA	2	12	0.881	0.955	0.905	Model/Sham
Model 8	Pos	PLS-DA	2	12	0.598	0.991	0.967	Model/Sham

**Table 2:** Change trends among the endogenous metabolites annotated.

No.	Annotated Metabolite	HMDB	Formula	Retention Time (min)	Molecular Weight	Adducts	Detected *m*/*z*	Model/Sham	NBH/Model
1	l-Arginine	HMDB0000517	C_6_H_14_N_4_O_2_	0.788	174.113	[M + H]^+^	175.0005	↓ ^b^	↑ ^c^
2	d-Maltose	HMDB0000163	C_12_H_22_O_11_	0.85	342.2965	[M + H]^+^	343.1235	↓ ^b^	↑ ^d^
3	Hypoxanthine	HMDB0000157	C_5_H_4_N_4_O	1.024	136.0427	[M − H]^−^	135.0352	↓ ^b^	↑ ^d^
4	Xanthine	HMDB0000292	C_5_H_4_N_4_O_2_	1.082	152.0354	[M + H]^+^	153.0418	↓ ^b^	↑ ^c^
5	L-Carnitine	HMDB0000062	C_7_H_16_NO_3_	1.09	162.1139	[M − H]^−^	160.8409	↓ ^a^	↑ ^c^
6	Oxidized glutathione	HMDB0003337	C_20_H_32_N_6_O_12_S_2_	1.13	612.1575	[M − H]^−^	611.1449	↓ ^b^	↑ ^c^
7	Niacinamide	HMDB0001406	C_6_H_6_N_2_O	1.15	122.0494	[M − H]^−^	121.9428	↓ ^b^	↑ ^d^
8	Adenine	HMDB0000034	C_5_H_5_N_5_	1.23	135.0558	[M + H]^+^	136.0635	↓ ^a^	↑ ^d^
9	Spermine	HMDB0001256	C_10_H_26_N_4_	1.24	202.2176	[M + H]^+^	203.9353	↓ ^b^	↑ ^c^
10	l-Methionine	HMDB0000696	C_5_H_11_NO_2_S	1.27	149.0524	[M + H]^+^	150.0596	↓ ^b^	↑ ^d^
11	l-Phenylalanine	HMDB0000159	C_9_H_11_NO_2_	2.64	165.0806	[M + H]^+^	166.0879	↑ ^b^	↓ ^d^
12	Pantothenic acid	HMDB0000210	C_9_H_17_NO_5_	3.9	219.1125	[M + H]^+^	220.1199	↓ ^b^	↑ ^d^
13	d-Histidine	HMDB0250763	C_6_H_9_N_3_O_2_	4.16	155.0709	[M + H]^+^	156.992	↓ ^b^	↑ ^d^
14	l-Glutamic acid	HMDB0000148	C_5_H_9_NO_4_	5.19	147.0545	[M + H]^+^	148.0617	↓ ^b^	↑ ^d^
15	d-Tryptophan	HMDB0013609	C_11_H_12_N_2_O_2_	5.52	204.0917	[M + Na]^+^	227.9845	↓ ^b^	↑ ^d^
16	Epsilon-caprolactam	HMDB0062769	C_6_H_11_NO	5.658	113.0825	[M + H]^+^	114.0842	↓ ^b^	↑ ^d^
17	Palmitic Acid	HMDB0000220	C_16_H_32_O_2_	12.36	256.4241	[M + Na]^+^	279.1611	↓ ^a^	↑ ^d^
18	valtrate	HMDB0034493	C_22_H_30_O_8_	15.055	212.1993	[M − H]^−^	211.0746	↓ ^b^	↑ ^d^
19	Phosphoenolpyruvic acid	HMDB0000263	C_3_H_5_O_6_P	15.06	168.0432	[M − H]^−^	167.3693	↓ ^a^	↑ ^c^
20	Argininosuccinic acid	HMDB0000052	C_10_H_18_N_4_O_6_	15.68	290.2731	[M + H]^+^	291.4325	↓ ^b^	↑ ^d^
21	Allantoic acid	HMDB0001209	C_4_H_8_N_4_O_4_	17.83	176.1307	[2M − H]^−^	351.2263	↓ ^b^	↑ ^d^
22	Eicosadienoic acid	HMDB0005060	C_20_H_36_O_2_	17.96	308.4986	[M − H]^−^	307.2645	↓ ^b^	↑ ^d^
23	Phthalic anhydride	HMDB0256501	C_8_H_4_O_3_	20.45	148.0174	[M + H]^+^	149.0252	↓ ^b^	↑ ^d^
24	Oleamide	HMDB0002117	C_18_H_35_NO	21.35	281.2741	[M − H]^−^	280.3808	↓ ^b^	↑ ^d^
25	Oxalacetic acid	HMDB0000223	C_4_H_4_O_5_	22.06	132.0824	[M − H]^−^	131.0426	↓ ^b^	↑ ^d^
26	Arachidonic acid	HMDB0001043	C_20_H_32_O_2_	22.29	304.2408	[M − H]^−^	303.2335	↓ ^b^	↑ ^d^
27	l-Tyrosine	HMDB0000158	C_9_H_11_NO_3_	22.57	181.0757	[M − H]^−^	180.9721	↓ ^b^	↑ ^c^
28	*N*-Hexadecanoylpyrrolidine	HMDB0032740	C_20_H_39_NO	23.558	309.3026	[M + H]^+^	310.8517	↓ ^b^	↑ ^d^
29	Stearoylethanolamide	HMDB13078	C_18_H_37_NO	23.713	327.5473	[M + H]^+^	328.9317	↓ ^a^	↑ ^c^
30	cis-Aconitic acid	HMDB0000461	C_6_H_6_O_6_	23.74	174.1082	[M − H]^−^	173.0821	↓ ^b^	↑ ^c^
31	Palmitic amide	HMDB0012273	C_16_H_33_NO	23.958	255.4393	[M + H]^+^	256.2741	↓ ^a^	↑ ^c^
32	2-Isopropyl-3-oxosuccinate	HMDB0012149	C_7_H_10_O_5_	24.05	174.1513	[2M + Na]^+^	371.3187	↑ ^b^	↓ ^d^
33	l-palmitoylcarnitine	HMDB0240774	C_23_H_45_NO_4_	24.44	399.6077	[M − H]^−^	398.3234	↓ ^b^	↑ ^d^
34	l-Glutamine	HMDB0000641	C_5_H_10_N_2_O_3_	24.52	146.0142	[M − H]^−^	145.6231	↓ ^b^	↑ ^d^

^a^ *p* < 0.05. ^b^ *p* < 0.01, Model group versus the Sham group. ^c^ *p* < 0.05. ^d^ *p* < 0.01, NBH group versus the Model group. “↓” denotes downregulated and “↑” denotes upregulated.

## Data Availability

All data are contained in the article ang Supplementary Material.

## Supplementary Materials

The following supporting information can be downloaded at: https://www.mdpi.com/article/10.3390/molecules28104064/s1, Figures S1 and S2.
