# Supplementary figures and images for: UPLC-QE-Orbitrap-Based Cell Metabolomics and Network Pharmacology to Reveal the Mechanism of N-Benzylhexadecanamide Isolated from Maca (Lepidium meyenii Walp.) against Testicular Dysfunction
Source: Molecules. 2023 May 12;28(10):4064. doi: 10.3390/molecules28104064 (PMC10222419; doi:10.3390/molecules28104064)

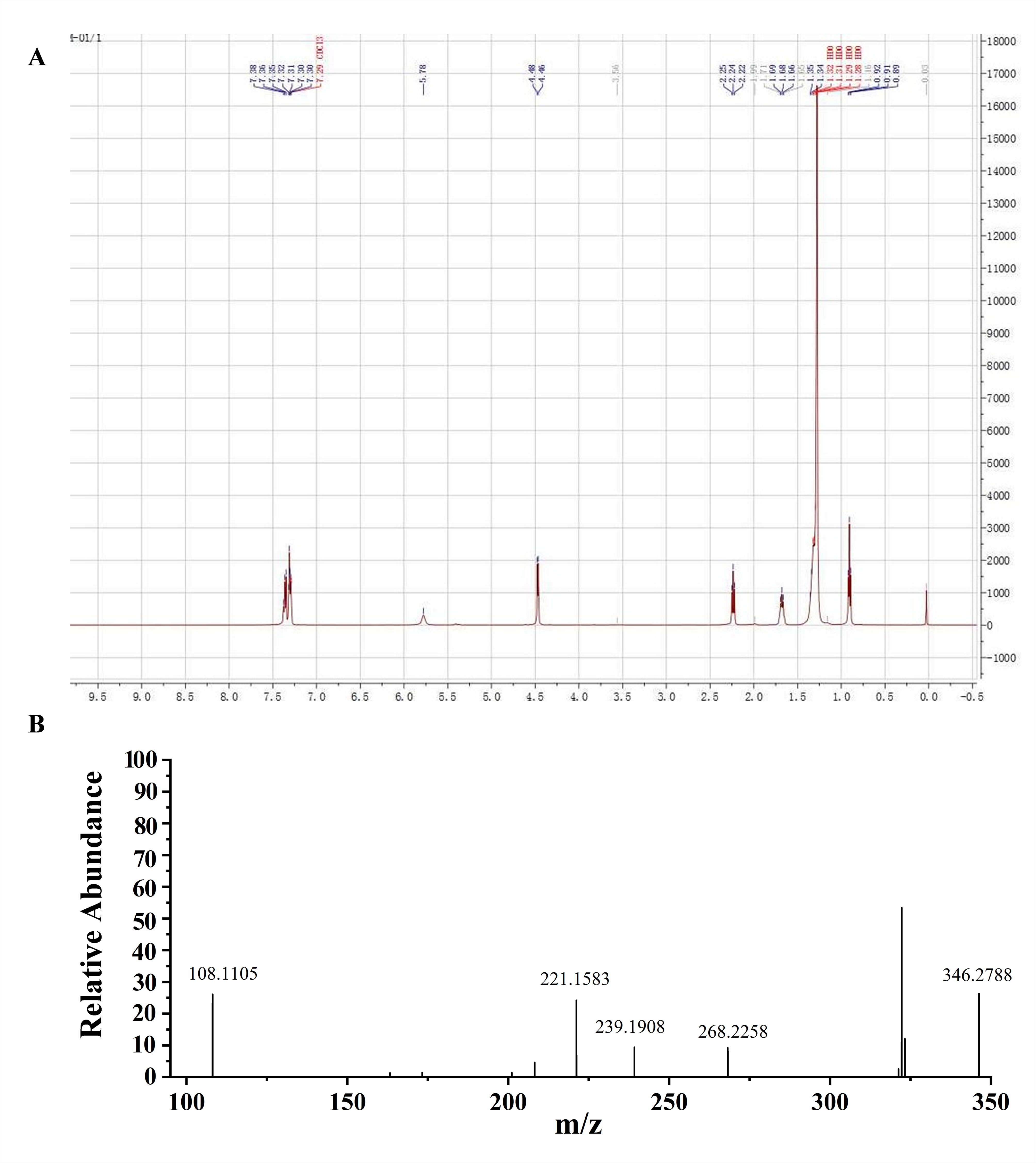

Supplement: Supplementary file 1 [file molecules-28-04064-s001.zip › Figure.S1.tif]

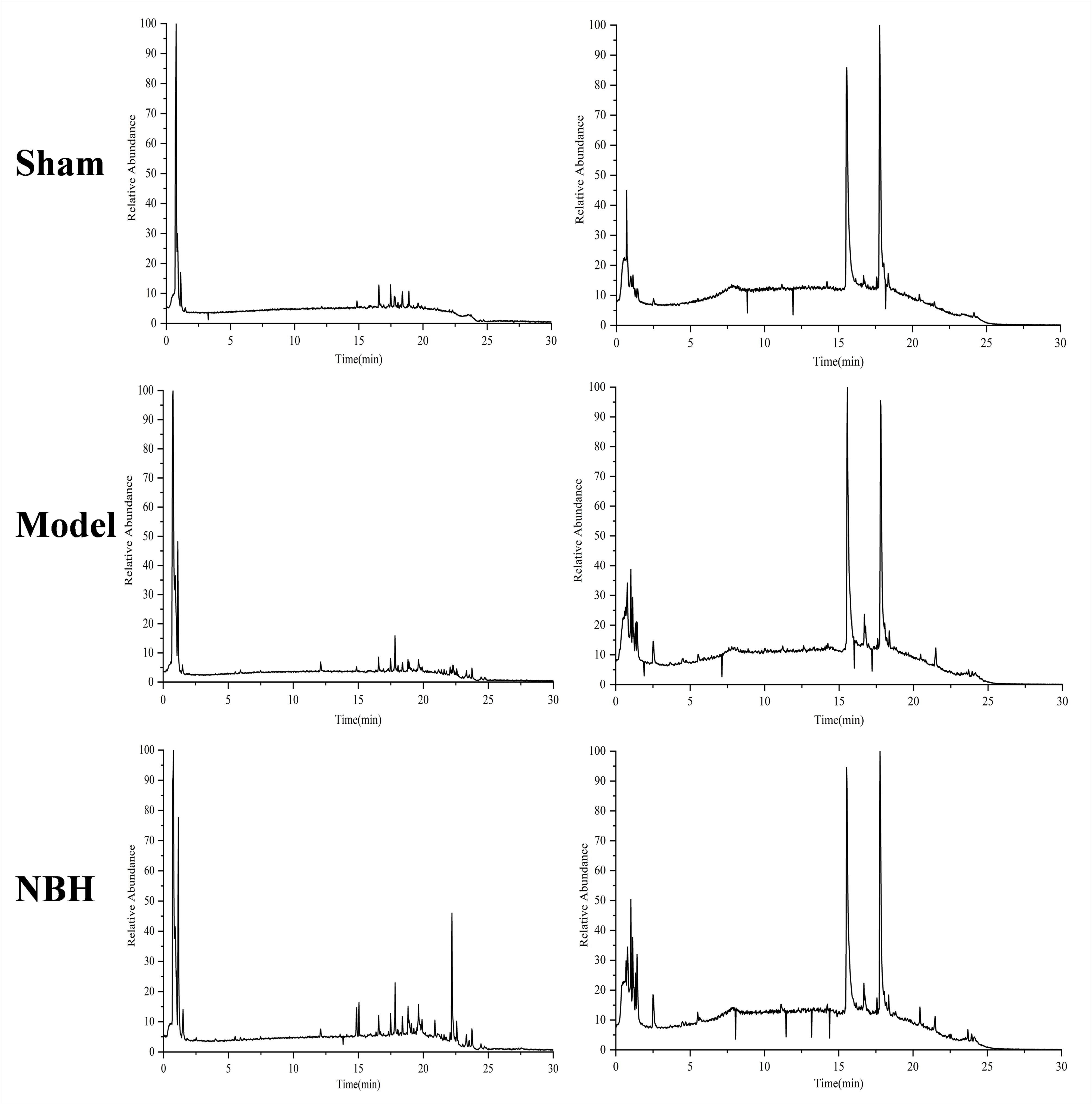

Supplement: Supplementary file 1 [file molecules-28-04064-s001.zip › Figure.S2.tif]
